# Acute resistance exercise induces Sestrin2 phosphorylation and p62 dephosphorylation in human skeletal muscle

**DOI:** 10.14814/phy2.13526

**Published:** 2017-12-21

**Authors:** Nina Zeng, Randall F. D'Souza, Vandre C. Figueiredo, James F. Markworth, Llion A. Roberts, Jonathan M. Peake, Cameron J. Mitchell, David Cameron‐Smith

**Affiliations:** ^1^ Liggins Institute The University of Auckland Private Bag 92019, Victoria Street West Auckland 1142 New Zealand; ^2^ Centre for Muscle Biology College of Health Sciences University of Kentucky Lexington Kentucky USA; ^3^ Department of Orthopedic Surgery University of Michigan Ann Arbor Michigan USA; ^4^ School of Allied Health Sciences & Menzies Health Institute Queensland Griffith University Gold Coast Australia; ^5^ Sport Performance Innovation and Knowledge Excellence, Queensland Academy of Sport Brisbane Australia; ^6^ School of Biomedical Sciences and Institute of Health and Biomedical Innovation Queensland University of Technology Brisbane Australia; ^7^ Food & Bio‐based Products Group AgResearch Palmerston North 4474 New Zealand; ^8^ Riddet Institute Palmerston North 4442 New Zealand

**Keywords:** Exercise training, p62^Ser403^, resistance exercise, sequestosome1, Sestrin, skeletal muscle

## Abstract

Sestrins (1, 2, 3) are a family of stress‐inducible proteins capable of attenuating oxidative stress, regulating metabolism, and stimulating autophagy. Sequestosome1 (p62) is also a stress‐inducible multifunctional protein acting as a signaling hub for oxidative stress and selective autophagy. It is unclear whether Sestrin and p62^Ser403^ are regulated acutely or chronically by resistance exercise (RE) or training (RT) in human skeletal muscle. Therefore, the acute and chronic effects of RE on Sestrin and p62 in human skeletal muscle were examined through two studies. In Study 1, nine active men (22.1 ± 2.2 years) performed a bout of single‐leg strength exercises and muscle biopsies were collected before, 2, 24, and 48 h after exercise. In Study 2, 10 active men (21.3 ± 1.9 years) strength trained for 12 weeks (2 days per week) and biopsies were collected pre‐ and post‐training. Acutely, 2 h postexercise, phosphorylation of p62^Ser403^ was downregulated, while there was a mobility shift of Sestrin2, indicative of increased phosphorylation. Forty‐eight hours postexercise, the protein expression of both Sestrin1 and total p62 increased. Chronic exercise had no impact on the gene or protein expression of Sestrin2/3 or p62, but Sestrin1 protein was upregulated. These findings demonstrated an inverse relationship between Sestrin2 and p62 phosphorylation after a single bout of RE, indicating they are transiently regulated. Contrarily, 12 weeks of RT increased protein expression of Sestrin1, suggesting that despite the strong sequence homology of the Sestrin family, they are differentially regulated in response to acute RE and chronic RT.

## Introduction

Physical activity, particularly resistance exercise (RE), is an intense muscle stressor that stimulates adaptive regulation of numerous nutrient and antioxidant‐sensitive pathways (Egan and Zierath 2013). Although mechanisms regulating muscle protein synthesis have been well studied (Dreyer et al. 2006), the complex signaling pathways regulating autophagy in response to RE (Fry et al. 2012) and RE‐induced oxidative stress (Çakır‐Atabek et al. 2015) remain elusive.

Sestrins are a family of stress‐inducible proteins that have multifunctional roles including attenuating oxidative stress, regulating mammalian target of rapamycin complex 1 (mTORC1), and stimulating autophagy (Lee et al. 2013). Mammals have three Sestrin genes (*SESN1/2/3*) that are regulated differently. Although Sestrin1 and 2 are regulated by p53, Sestrin3 is regulated by forkhead box O (FOXO) (Parmigiani and Budanov 2016). Sestrin2 shows intrinsic oxidoreductase activity (Budanov et al. 2004); however, it was later shown that this is not required for its antioxidant functioning (Woo et al. 2009). In circumstances of increased oxidative stress, Sestrin1 and 2 prevent reactive oxygen species (ROS) accumulation by inducing selective autophagic degradation of Kelch‐like ECH‐associated protein (Keap1), an inhibitor of the nuclear factor (erythroid‐derived 2)‐like 2 (Nrf2), thereby upregulating Nrf2‐dependent antioxidant gene transcription (Bae et al. 2013). Sestrin2 has also been proposed as a leucine sensor (Parmigiani et al. 2014; Wolfson et al. 2016) and in vitro analysis has identified it as a phosphoprotein, which in response to leucine deprivation, is phosphorylated and interacts with GTPase‐activating protein activity toward Rags 2 (GATOR2) to inhibit mTORC1 activity (Kim et al. 2015; Kimball et al. 2016). Sestrin3, however, is upregulated in the skeletal muscle of type 2 diabetic patients (Nascimento et al. 2013) and maintains insulin sensitivity in overfed mice via protein kinase B (Tao et al. 2015).

p62 is a stress‐inducible protein involved in oxidative stress and autophagic clearance of polyubiquitinated proteins (Katsuragi et al. 2015). Autophagy is an evolutionarily conserved process that recycles protein aggregates and malfunctioning organelles. Macroautophagy, microautophagy, and chaperone‐mediated autophagy are the three main forms of autophagy (Tanida 2011). p62 plays an important role in selective macroautophagic protein degradation. It binds to ubiquitinated proteins and microtubule‐associated protein 1 light chain 3 (LC3), allowing it to recruit these proteins to autophagosomes, which fuse with lysosomes for protein degradation (Lamark et al. 2009). It has been shown that phosphorylation of p62 at Serine 403 (Ser403) plays a critical role in selective macroautophagy, because phosphorylating p62^Ser403^ stabilizes the association between p62 and ubiquitinated protein, which enables efficient autophagosome formation (Matsumoto et al. 2011).

Included in the complex functionality of Sestrin2 is its interaction with p62 (Ro et al. 2014). In vitro, Sestrin2 associates with p62 and Unc‐51‐like protein kinase 1 (ULK1), forming a complex that induces ULK1 to phosphorylate p62^Ser403^ (Ro et al. 2014). Phosphorylated p62^Ser403^ enhances its binding affinity to Keap1 (Matsumoto et al. 2011), thereby initiating autophagosome formation around the cargos, which ultimately leads to selective autophagic degradation of Keap1, hence freeing Nrf2 and enabling its translocation to the nucleus to upregulate antioxidant gene expression (Ichimura et al. 2013).

Sestrins are also critical regulators of muscle aging (Budanov et al. 2010). Genetic ablation of *Drosophila* Sestrin (dSesn) induces the early onset of skeletal muscle degeneration and accumulated defective mitochondria (Lee et al. 2010). Resistance training (RT) is one of the most important strategies to prevent muscle wastage (Sanchis‐Gomar et al. 2011); however, no studies have assessed the effects of RT on Sestrin in human. To date, there is only evidence of endurance exercise increasing the protein expressions of Sestrin2 and 3 in mouse skeletal muscle which occurs in conjunction with an increase in autophagy (Liu et al. 2015; Lenhare et al. 2017). Whether the three mammalian Sestrin proteins differentially control skeletal muscle function and which plays a more important role on human muscle health is unclear. Similarly, it remains unknown whether Sestrin and p62^Ser403^ are regulated acutely or chronically by RE and RT, respectively, in human skeletal muscle. Therefore, this study aimed to measure how acute RE affects Sestrin2 and p62^Ser403^ phosphorylation and examined the effects of RE on the protein and mRNA expression of Sestrin paralogs. Separately, the chronic effects of 12 weeks of RT on Sestrin protein and mRNA expression were also investigated.

## Materials and Method

### Ethics approval

All participants were informed of the requirements and potential risks of the studies prior to providing their written informed consent. The experimental procedures adhered to the standards set by the latest version of the Declaration of Helsinki and were approved by the Human Research Ethics Committee of The University of Queensland.

### Study design

Subjects in this study were a subset of a larger trial (Roberts et al. 2015). In both studies, all participants had at least 12 months of experience in strength training and were familiar with all exercises used in the studies. In the acute study, nine physically active trained men (22.1 ± 2.2 years old) completed a bout of single‐leg strength exercise. Eight repetitions maximum (RM) strength of unilateral knee extension (71 ± 12.0 kg) and unilateral 45° leg press (299 ± 44.8 kg) for both legs were assessed 4–5 days prior to experimental exercise bout. At the same time, familiarization for the single‐leg squats and walking lunge exercise were performed. On the day of the trial, the RE bout included six sets of 45° leg press and knee extensions at 8, 8, 10, 12, 10, and 10 RM, and three sets of single‐leg squats and walking lunges at 12 RM.

In the chronic study, 10 trained men (21.3 ± 1.9 years old) participated in a 12‐week lower body RT program with training twice a week, separated by 72 h. Muscle strength for training load prescription was assessed 10–14 days before the first training session. Bilateral 45° leg press (348 ± 80 kg), knee extension (88 ± 9 kg), and knee flexion (75 ± 11 kg) 1 RMs were determined. For the training session, the loads were set to include fatigue at 8, 10, and 12 RM and weights for walking lunges corresponded to a proportion of each participant's pretraining body mass (PTBM) (79.2 ± 4.4 kg). Each training session was approximately 45 min and included six sets of 45° leg press at 8, 8, 10, 12, 10, and 10 RM, and three sets of knee extension and flexion at 12 RM. Three sets of walking lunges were also performed with week 1–3 having 20% of PTBM, and an additional 5 kg added progressively every 3 weeks. Additionally, three sets of plyometrics exercises comprising countermovement drop jumps, slow eccentric squat jumps, split lunge jumps, and countermovement box jumps were performed at 50% of lunge load. In both studies, after each exercise session, participants completed active recovery by cycling on a stationary bicycle at a low, self‐selected intensity for 10 min.

To control for postexercise diet, in the acute study, participants consumed a standardized meal 2 h before the pre‐exercise biopsy and consumed 30 g of whey protein before the recovery period. The participants then fasted until the 2 h biopsy, after which they consumed another 30 g of whey protein. Muscle biopsies from the vastus lateralis were collected before, 2, 24, and 48 h postexercise. In the chronic study, biopsies were collected 4–5 days before the first training session and post‐training biopsies were collected 6–7 days after the last training session in a fasted state. All muscle samples were snap frozen in liquid nitrogen and stored at −80°C until further analysis.

### Western blotting

Twenty‐five milligrams of muscle biopsies were homogenized with RIPA lysis buffer (Millipore, Temecula, CA) with added Halt™ protease and phosphatase inhibitor cocktail (Thermo Fisher Scientific, MA). After centrifugation, supernatants were collected and total protein concentration was determined using the Pierce™ BCA Protein Assay Kit (Thermo Fisher Scientific). Equal amounts of protein were boiled in Laemmli buffer at 95°C for 5 min. 20 *μ*g of protein was separated by SDS‐PAGE and transferred to PVDF membranes (Bio‐Rad Laboratories, Inc., CA) using the semidry Trans‐Blot Turbo™ device (Bio‐Rad). Membranes were incubated with the following primary antibodies, total p62, Sestrin1 and 3 (Abcam, ab56416, ab103121, and ab97792, respectively), Sestrin2 (ProteinTech, 10795‐1‐AP), and p62^Ser403^ (GeneTex, GTX128171) (all at 1:1000 dilution, except Sestrin1 which is at 1:100) overnight, and the appropriate anti‐rabbit or anti‐mouse secondary antibodies (Jackson ImmunoResearch Laboratories, PA) linked to horseradish peroxidase (1:10,000) for 1 h at room temperature. The membranes were exposed on a ChemiDoc image device (Bio‐Rad) using enhanced chemiluminescence reagent (ECL Select kit; GE Healthcare Ltd., Little Chalfont, UK). Bands were quantified using ImageJ software (NIH, Bethesda, MD). Western blot data were normalized to the housekeeping protein GAPDH (Abcam, ab36840) (1: 10,000).

### Sestrin2 electrophoretic mobility

To allow for resolution of Sestrin2 into multiple electrophoretic forms as previously demonstrated in Kimball et al. (2016), samples were electrophoresed through 8% polyacrylamide gels (acrylamide‐*bis*‐acrylamide, 19:1). When human embryonic kidney cells (HEK293) were incubated in complete medium, Sestrin2 separated into three bands: *α*,* β*, and *γ*. However, when incubated in leucine‐deficient medium, there was a mobility shift of the protein, resulting in the appearance of a slower migrating *δ* band (Kimball et al. 2016). To provide evidence that the multiple electrophoretic bands represented different phosphorylated forms of Sestrin2, Kimball et al. treated samples with lambda protein phosphatase, which led to a shift in the migration of Sestrin2 into a single band, suggesting additional bands represented multiple phosphorylated forms of the protein. Mass spectrometry analysis of immunoprecipitates of endogenous Sestrin2 further confirmed it as a phosphoprotein as three phosphorylation sites, Thr232, Ser249, and Ser279, were identified (Kimball et al. 2016). In the present study, to measure the intensity of Sestrin2 phosphorylation, the abundance of the slowest migrating *δ* form of Sestrin2 was taken as phosphorylation. It is known that phosphorylation results in the protein migrating at a higher, apparent molecular mass (Wegener and Jones 1984; Peck 2006). Total Sestrin2 protein was recorded as the expression of all forms of Sestrin2 (*δ*,* γ*,* β*, and *α* form).

### RNA extraction and quantitative real‐time PCR

Following the manufacturer's instructions from the AllPrep^®^ DNA/RNA/miRNA Universal Kit (Qiagen GmbH, Hilden, Germany), total RNA was extracted from 20 mg of muscle biopsies. 1500 ng of input RNA was then used for cDNA synthesis using High‐Capacity RNA‐to‐cDNA™ kit (Life Technologies, Carlsbad, CA). Messenger RNA (mRNA) was measured by RT‐PCR on a LightCycler 480 II (Roche Applied Science, Penzberg, Germany) using SYBR Green I Master Mix (Roche Applied Science). Target mRNAs were *SESN1*,* SESN2*,* SESN3*, and *p62*. Primers were designed using BLAST software (Ye et al. 2012) with sequences in Table 1. Relative fold changes were determined using the 2^−ΔΔCT^ method (Schmittgen and Livak 2008). To compare the basal expression levels of the different Sestrin paralogs, 2^−ΔCT^ was used. The geometric mean of three reference genes was used for normalization (Vandesompele et al. 2002). The recently proposed human reference genes (Eisenberg and Levanon 2013), chromosome 1 open reading frame 43 (*C1orf43*), charged multivesicular body protein 2A (*CHMP2A*), and ER membrane protein complex subunit 7 (*EMC7*) were identified as the least variable and used as reference genes (Table 1).

**Table 1:** mRNA sequences

Gene	Sequence
*CHMP2A* (forward)	CGCTATGTGCGCAAGTTTGT
*CHMP2A* (reverse)	GGGGCAACTTCAGCTGTCTG
*C1orf43* (forward)	CTATGGGACAGGGGTCTTTGG
*C1orf43* (reverse)	TTTGGCTGCTGACTGGTGAT
*EMC7* (forward)	GGGCTGGACAGACTTTCTAATG
*EMC7* (reverse)	CTCCATTTCCCGTCTCATGTCAG
*SESN1* (forward)	TTTCGTGTCCAGGACTATTGC
*SESN1* (reverse)	ACTGTCCCACATCTGGATAAAGG
*SESN2* (forward)	CAACCTCTTCTGGAGGCACTT
*SESN2* (reverse)	CCTGCTCAGGAGTCAGGTCA
*SESN3* (forward)	CAGGCAGCAACTTTGGGATTGT
*SESN3* (reverse)	AGACGCCTCTTCATCTTCCCTT
*p62* (forward)	GAATCAGCTTCTGGTCCATCGG
*p62* (reverse)	GCTTCTTTTCCCTCCGTGCT

Forward and reverse sequences of analyzed genes.

### Statistical analysis

To measure differences across time in the acute study, one‐way repeated measures analysis of variance (ANOVA) was performed using SigmaPlot (Systat 218 Software Inc., San Jose). Holm–Sidak post hoc tests were used where appropriate to compare postexercise values to baseline with significance set at *P* < 0.05. For the chronic study, time differences were conducted using a paired Student's *t* test. To compare the basal differences of all three Sestrins, one‐way ANOVA was used. All values are presented as means ± SEM.

## Results

### Acute exercise

**Figure 1:**
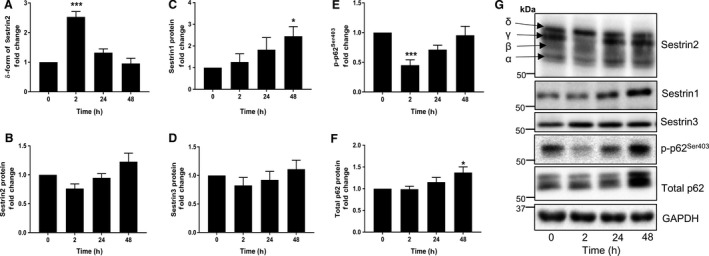
Effects of acute resistance exercise on Sestrin and p62 protein. The relative abundance of Sestrin2 in *δ* form (A), total Sestrin2 protein (B), Sestrin1 protein (C), Sestrin3 protein (D), phosphorylation status of p62^Ser403^ (E), and total p62 protein (F) following acute RE. Representative western blots (G). Data are expressed as fold change from rest and error bars represent SEM. *P < 0.05 and ***P < 0.001 versus respective baseline samples.

**Figure 2:**
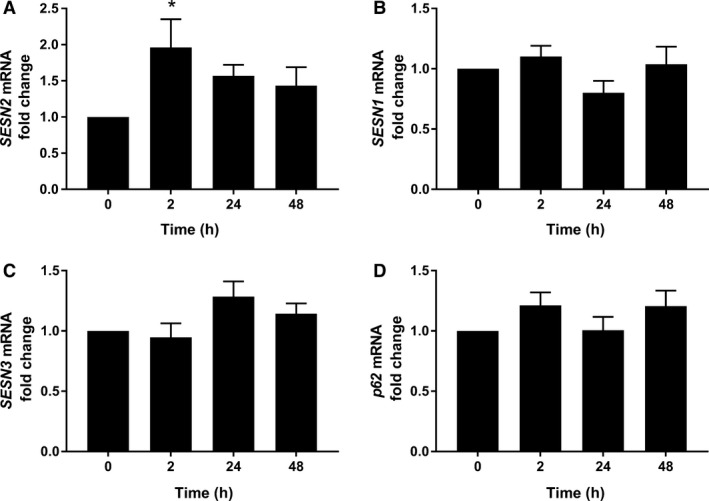
Effects of acute resistance exercise on mRNA expression. The mRNA expression of *SESN2* (A), *SESN1* (B), *SESN3* (C), and *p62* (D). Data are expressed as fold change from rest and error bars represent SEM. *P < 0.05 versus respective baseline samples.

**Figure 3:**
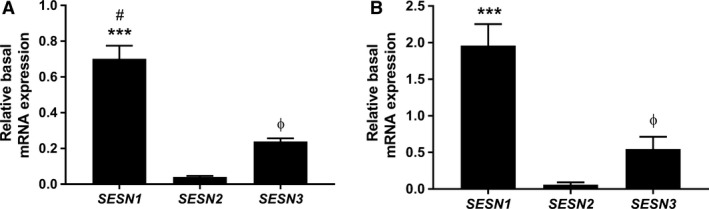
Basal expression of Sestrin paralogs. The basal mRNA expression of *SESN1*,* 2*,* 3* in the acute (A) and chronic (B) study. Data are expressed as means ± SEM. ***Difference between *SESN1* and *SESN2* (*P* < 0.001). ^#^Difference between *SESN1* and *SESN3* (*P* < 0.05). ^Ф^Difference between *SESN3* and *SESN2* (*P* < 0.05).

Sestrin2 phosphorylation (assessed by the mobility shift of the *δ* band) was higher 2 h after exercise (*P* < 0.001) (Fig. 1A), whereas the phosphorylation of p62^Ser403^ was reduced 2 h after exercise (*P* < 0.001) (Fig. 1E). No difference was observed in the total protein expression of Sestrin2 (Fig. 1B), but its mRNA expression (*SESN2*) increased 2 h postexercise (*P* = 0.015) (Fig. 2A). There were no changes in the mRNA expressions of *SESN1*,* SESN3*, and *p62* (Fig. 2B–D), and protein expression of Sestrin3 (Fig. 1D). However, the protein expression of Sestrin1 and total p62 increased 48 h postexercise (*P* = 0.025 and *P* = 0.031, respectively) (Fig. 1C and F). Basal mRNA expression of *SESN1* was significantly more abundant than *SESN2* (*P* < 0.001) or *SESN3* (*P* = 0.043). *SESN3* was also more abundant than *SESN2* (*P* = 0.043) (Fig. 3A).

### Chronic exercise

**Figure 4:**
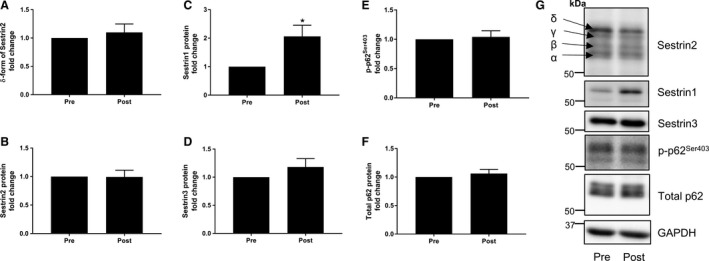
Effects of chronic resistance training on Sestrin and p62 protein. The relative abundance of Sestrin2 in *δ* form (A), total Sestrin2 protein (B), Sestrin1 protein (C), Sestrin3 protein (D), phosphorylation status of p62^Ser403^ (E), and total p62 protein (F). Representative western blots (G). Data are expressed as fold change from rest and error bars represent SEM. *P < 0.05 versus respective baseline samples.

The phosphorylation states of Sestrin2 and p62 were unchanged following RT (Fig. 4A and E). Also, no changes in protein and mRNA expressions of Sestrin2, Sestrin3, and total p62 were observed (Fig. 4B, D, and F). However, Sestrin1 protein was increased with RT (*P* = 0.026) (Fig. 4C). Similar to the acute study, prior to training, the mRNA expression of *SESN1* was significantly more abundant than *SESN2* (*P* < 0.001). *SESN3* was also more abundant than *SESN2* (*P* = 0.05) (Fig. 3B).

## Discussion

The present study confirmed the role of Sestrin2 as a phosphoprotein (Ro et al. 2014; Kimball et al. 2016; Nikonorova et al. 2017) and extended previous findings to show it is responsive to acute RE in human skeletal muscle. There was an electrophoretic mobility shift resulting in increased abundance of a slower migrating *δ* band of Sestrin2, indicative of increased phosphorylation acutely following RE. Mirroring the time course of increased Sestrin2 phosphorylation, p62^Ser403^ phosphorylation was transiently downregulated following RE. After 12 weeks of RT, resting total protein abundance and basal phosphorylation of Sestrin2 and p62 were unaltered. However, there was an increased Sestrin1 protein abundance, suggesting that despite the strong sequence homology of the Sestrin family, they are differentially regulated in response to RE and RT.

### Effect of acute exercise

Following RE, mRNA expression of *SESN2* increased 2 h postexercise. Exposure of primary human myotubes to reactive oxygen species (H_2_O_2_) for 6 h also increased mRNA expression of *SESN2* (Nascimento et al. 2013). Although RE is primarily an anabolic stimulus, it has been shown to be a potent inducer of acute oxidative stress (Polotow et al. 2017). Sestrins protect cells from oxidative stress and cellular damage, as their repression upregulated ROS production and induced genetic instability (Kopnin et al. 2007). The upregulation of *SESN2* postexercise may be an adaptation to protect skeletal muscle cells from exercise‐induced oxidative stress.

There was also an increase in the relative amount of Sestrin2 present in the heavier *δ* band at 2 h, returning to pre‐exercise levels by 24 h. Sestrin2 has been proposed as a leucine sensor (Parmigiani et al. 2014; Wolfson et al. 2016). In HEK293, increased leucine concentrations in the media resulted in a reduction of the relative amount of the *δ* band, which promoted mTORC1 activation, as assessed by an increase in phosphorylation of p70S6K1^Thr389^ (Kimball et al. 2016). In this study, participants consumed 30 g of whey protein before the recovery period. A reduction of the *δ* band should be expected; however, no inverse relationship between Sestrin2 phosphorylation and mTORC1 activation was observed. Conversely, the intensity of the *δ* band increased and p70S6K1^Thr389^ was highly phosphorylated 2 h postexercise as demonstrated previously (Roberts et al. 2015). The discrepancy could be due to different tissue types and stimuli, as exercise is an intense muscle stressor that affects multiple pathways (Egan and Zierath 2013). Furthermore, there is limited evidence demonstrating action of leucine on Sestrin function in cells other than HEK293 and mouse fibroblasts (Chantranupong et al. 2014; Parmigiani et al. 2014; Wolfson et al. 2016). Thus, the nature of Sestrin2 leucine sensor properties is still under debate (Lee et al. 2016; Saxton et al. 2016). Therefore, future studies should aim to separate feeding or exercise stimuli with the aim of providing more insight into the possible in vivo functioning of Sestrin2 phosphorylation.

In contrast to Sestrin2 phosphorylation, p62^Ser403^ phosphorylation was repressed 2 h postexercise. Under in vitro conditions, the association between Sestrin2, p62, and ULK1 promotes ULK1‐mediated p62^Ser403^ phosphorylation, resulting in selective degradation of polyubiquitinated cargos, such as Keap1 (Matsumoto et al. 2011; Ro et al. 2014). Degradation of Keap1 allows Nrf2 to be translocated to the nucleus to upregulate antioxidant gene expression (Ichimura et al. 2013). The acute postexercise dephosphorylation of p62^Ser403^ observed in the current study could be suggestive of diminished ubiquitin‐mediated selective macroautophagic protein degradation. Furthermore, in agreement with the current study, RE has been shown to upregulate total p62, 24 and 48 h following exercise (Ogborn et al. 2015), indicating that acute RE might be suppressing macroautophagy, as p62 accumulates when autophagy is inhibited (Mizushima et al. 2010). Additionally, by measuring the conversion of cytosolic microtubule‐associated protein 1 (LC3B‐I) to the autophagosomal membrane‐associated form, LC3B‐II, a marker of enhanced autophagy, it was demonstrated that macroautophagy was depressed in both young and old adults following an acute bout of RE (Glynn et al. 2010; Fry et al. 2012).

p62 has been found to be induced at the transcriptional level by ROS under cellular stress (Jain et al. 2010), therefore to evaluate the role of Sestrin2 and p62 in regulating oxidative stress, future studies should explore oxidative stress markers, Keap1 degradation, Nrf2 upregulation, and antioxidant response. As exercise influences multiple pathways, the present data does not allow for strong mechanistic conclusions regarding the role of Sestrin2 phosphorylation in regulating p62^Ser403^ phosphorylation and its implication in redox homeostasis and selective autophagy. Due to limited available muscle tissues, coimmunoprecipitation analyses were not undertaken, making it unclear whether there was a functional association between Sestrin2 and p62. Furthermore, a limitation of the study was a lack of control for feeding at the 24 and 48 h biopsies collections, which could have affected the result observed at these time points. Future studies should investigate the physical association between Sestrin2 and p62 and control for feeding at all time points. The present study demonstrated that following RE, a clear inverse relationship between the phosphorylation status of Sestrin2 and p62^Ser403^ exists, and they are transiently regulated after RE, which may play a role in cellular adaptation in human skeletal muscle.

### Effect of chronic exercise

Skeletal muscle is sensitive to both acute and chronic stresses associated with RE and RT. The mechanisms associated with the acute transient response to RE might be different compared to chronic adaptation which are measured in the rested state, since these responses are influenced by multiple factors including frequency of exercise, recovery period, and training history of individuals (Abernethy et al. 1994). Literature on the relationship of Sestrin and exercise is limited. The current understanding is a single bout of aerobic exercise increased Sestrin2 protein in mice (Lenhare et al. 2017), while long‐term endurance exercise increased the protein expression of Sestrin2 and 3 and basal level of muscle autophagy (Liu et al. 2015). In this study, long‐term RT for 12 weeks did not alter the protein or mRNA expression of Sestrin2, 3, and total p62, or the basal phosphorylation status of Sestrin2 and p62^Ser403^. This could be due to the use of different exercise protocol, since different modes of exercise produce distinct myofiber adaptations, while RT increases strength and muscle fiber cross‐sectional area, endurance exercise improves oxidative metabolism by increasing mitochondrial content and capillary densities (Wilkinson et al. 2008). Interestingly, the protein expression of Sestrin1 increased significantly following RT and also 48 h following RE. However, direct comparison between the acute and chronic effects on Sestrin1 cannot be made, since fasting biopsies were collected in the present chronic study, while in the acute study, biopsies were collected in the fed state.

In agreement with a previous study, compared with Sestrin2 and 3, Sestrin1 is more abundantly expressed in skeletal muscle (Peeters et al. 2003). Silencing of Sestrin1 in human embryonic fibroblasts inhibited cell proliferation and accelerated cell senescence due to excess ROS production (Budanov et al. 2004). Moreover, studies from drosophila and mouse models provided a connection between Sestrins and muscle growth, as knockout of Sestrin resulted in muscle degeneration (Lee et al. 2010). Additionally, silencing Sestrin3 in human myotubes increased myostatin expression, which is a negative regulator of muscle growth (Nascimento et al. 2013). Recruited subjects demonstrated an increase in both strength and muscle mass after 12 weeks of training (Roberts et al. 2015). Taken together, these observations suggest a potential link between Sestrin1 and the regulation of cell growth, which warrants further investigation to clarify the distinct roles played by each Sestrin family members in human skeletal muscle.

## Conclusion

Sestrin and p62 are multifunctional proteins involved in many cellular processes, including suppressing oxidative stress, mTORC1, and autophagy regulation (Katsuragi et al. 2015; Parmigiani and Budanov 2016). The present analysis demonstrated that while Sestrin family members share considerable sequence homology, each is regulated independently in response to RE. Sestrin3 was not affected by RE, whereas long‐term training induced the protein expression of Sestrin1. In response to RE, there was a transient mobility shift of Sestrin2, indicative of increased phosphorylation. Mirroring this response, p62^Ser403^ phosphorylation was downregulated. It appears that both Sestrin2 and p62^Ser403^ are transiently regulated, and may be functionally involved in the adaptive regulatory mechanisms elicited by human skeletal muscle after intense RE.

## Conflict of Interest

None declared.
